# Socially Assistive Robots for Dementia Care: A Human-in-the-Loop Approach to Person-Centered Care Protocols

**DOI:** 10.1093/geroni/igaf122.1543

**Published:** 2025-12-31

**Authors:** Jing Wang, Sajay Arthanat, Momotaz Begum, Ola Ghattas, Marzan Alam, Moniruzzaman Akash, Dain LaRoche

**Affiliations:** University of New Hampshire, Durham, New Hampshire, United States; University of New Hampshire, Durham, New Hampshire, United States; University of New Hampshire, Durham, New Hampshire, United States; University of New Hampshire, Durham, New Hampshire, United States; University of New Hampshire, Durham, New Hampshire, United States; University of New Hampshire, Durham, New Hampshire, United States; University of New Hampshire, Durham, New Hampshire, United States

## Abstract

The integration of Socially Assistive Robots (SARs) in dementia care offers innovative solutions to support aging in place and enhance autonomy for persons living with dementia (PLWD). This study explores the collaborative development of SAR care protocols using human-in-the-loop (HITL) and person-centered design approaches to support PLWD and their care partners in home settings. Grounded in interdisciplinary discussions between robotics scientists and health scientists, notes from the care protocol tailoring meetings with participants, and ongoing pilot study deploying SARs in real-world home environments with five caregiver-care recipient dyads over six months, this study highlights how direct user involvement and iterative refinement enhance SAR functionality and usability. Key design modifications included enhanced voice interaction, simplified task reminders, and personalized care routines, ensuring that SARs remained intuitive and responsive to cognitive and sensory limitations. To improve usability, the docking system was redesigned to function in various lighting conditions, while a tablet interface was incorporated for interactive guidance. User feedback was crucial in optimizing medication adherence prompts, mobility assistance, and social engagement features. Findings emphasize the importance of collaborative, person-centered refinement to create adaptive robotic systems that align with PLWD preferences and caregiving workflows. This study offers insights into real-world SAR deployment and provides a framework for integrating user-centered iterative design into assistive technology development. Future research should focus on scalability, ethical considerations, and long-term engagement to maximize SAR effectiveness in dementia care.

